# Simultaneous EEG-fMRI during a Working Memory Task: Modulations in Low and High Frequency Bands

**DOI:** 10.1371/journal.pone.0010298

**Published:** 2010-04-22

**Authors:** Lars Michels, Kerstin Bucher, Rafael Lüchinger, Peter Klaver, Ernst Martin, Daniel Jeanmonod, Daniel Brandeis

**Affiliations:** 1 Functional Neurosurgery, University Hospital Zürich, Zürich, Switzerland; 2 MR-Center, University Children's Hospital, Zürich, Switzerland; 3 Department of Child and Adolescent Psychiatry, University of Zürich, Zürich, Switzerland; 4 Zürich Center for Integrative Human Physiology (ZIHP), Zürich, Switzerland; 5 Department of Child and Adolescent Psychiatry and Psychotherapy, Central Institute of Mental Health, Mannheim, Germany; Ecole Polytechnique Fédérale de Lausanne, Switzerland

## Abstract

**Background:**

EEG studies of working memory (WM) have demonstrated load dependent frequency band modulations. FMRI studies have localized load modulated activity to the dorsolateral prefrontal cortex (DLPFC), medial prefrontal cortex (MPFC), and posterior parietal cortex (PPC). Recently, an EEG-fMRI study found that low frequency band (theta and alpha) activity negatively correlated with the BOLD signal during the retention phase of a WM task. However, the coupling of higher (beta and gamma) frequencies with the BOLD signal during WM is unknown.

**Methodology:**

In 16 healthy adult subjects, we first investigated EEG-BOLD signal correlations for theta (5–7 Hz), alpha1 (8–10), alpha2 (10–12 Hz), beta1 (13–20), beta2 (20–30 Hz), and gamma (30–40 Hz) during the retention period of a WM task with set size 2 and 5. Secondly, we investigated whether load sensitive brain regions are characterised by effects that relate frequency bands to BOLD signals effects.

**Principal Findings:**

We found negative theta-BOLD signal correlations in the MPFC, PPC, and cingulate cortex (ACC and PCC). For alpha1 positive correlations with the BOLD signal were found in ACC, MPFC, and PCC; negative correlations were observed in DLPFC, PPC, and inferior frontal gyrus (IFG). Negative alpha2-BOLD signal correlations were observed in parieto-occipital regions. Beta1-BOLD signal correlations were positive in ACC and negative in precentral and superior temporal gyrus. Beta2 and gamma showed only positive correlations with BOLD, e.g., in DLPFC, MPFC (gamma) and IFG (beta2/gamma). The load analysis revealed that theta and—with one exception—beta and gamma demonstrated exclusively positive load effects, while alpha1 showed only negative effects.

**Conclusions:**

We conclude that the directions of EEG-BOLD signal correlations vary across brain regions and EEG frequency bands. In addition, some brain regions show both load sensitive BOLD and frequency band effects. Our data indicate that lower as well as higher frequency brain oscillations are linked to neurovascular processes during WM.

## Introduction

Functional magnetic resonance imaging (fMRI) studies have identified working memory (WM) related activities in a widely-distributed network involving the medial prefrontal cortex (MPFC), dorsolateral prefrontal cortex (DLPFC), posterior parietal cortex (PPC), hippocampus, insula, and occipito-temporal cortex [1], [2], [3], [4], [5], [6], [7], [8], [9], [10], [11], [12]. In addition, electroencephalography (EEG) and magnetoencephalography (MEG) studies have demonstrated that MPFC theta (4–8 Hz) power increases with the number of items retained in memory [5], [13], [14], [15], [16], [17]. In the alpha band (8–13 Hz), some EEG/MEG studies reported an increase of spectral power in parieto-occipital areas (including the precuneus) with load [4], [18], [19], [20], whereas others found decreased [8], [14], [21] or both increased and decreased [14], [22] activity in these regions.

To date, only few EEG-fMRI studies of WM examined the relationship between EEG band dynamics and fMRI responses, i.e. the Blood Oxygen Level Dependency (BOLD) signal, during the retention period of a WM task [22], [23], [24]. A recent simultaneous EEG-fMRI study used an independent component analysis (ICA) approach to first extract the EEG power of one frontal theta and one alpha band component, which was then correlated with the BOLD signal of the retention period [23]. These authors found negative theta-BOLD signal correlations in the ACC, PCC, MPFC and PPC but not in the DLPFC. For alpha, the authors reported negative correlations in the MPFC, primary visual cortex, and middle temporal gyrus. However, none of the above mentioned studies reported BOLD signal correlations with higher frequency bands such as beta (>13 Hz) or gamma (>30 Hz). Yet, it has been demonstrated that the power of local field potential (LFP) oscillations in the gamma range and the BOLD response show positive correlations in animals [25], [26] during visual stimulation. Moreover, human beta and gamma demonstrate predominantly positive load effects during different WM tasks [9], [27], [28], [29], [30], [31], [32], [33], [34], [35], suggesting that these frequencies might show a positive interaction with the BOLD signal.

In the present study we first examined load independent EEG-BOLD signal correlations during the retention period for low as well as for high frequency bands. In the same time window we performed a load sensitive analysis (BOLD contrast: set size 5> set size 2) to investigate whether regions related to WM are further characterised by frequency band related load effects.

We hypothesized that during the retention period theta and alpha would mainly show negative correlations with the BOLD signal as previously reported, and that beta and gamma exhibit positive correlations. We further expect that load sensitive WM areas should demonstrate both BOLD and frequency band related load effects.

## Materials and Methods

### Subjects

Sixteen healthy volunteers (mean age 24.8±3.8 years, 8 females) participated in this study. All subjects were right-handed and had normal or corrected-to-normal vision. The study falls under the ethical approval of the ‘Kantonale Ethikkommission Zürich’ (http://www.kek.zh.ch). All participants gave written informed consent prior to participation.

### Task and procedure

**Figure 1:**
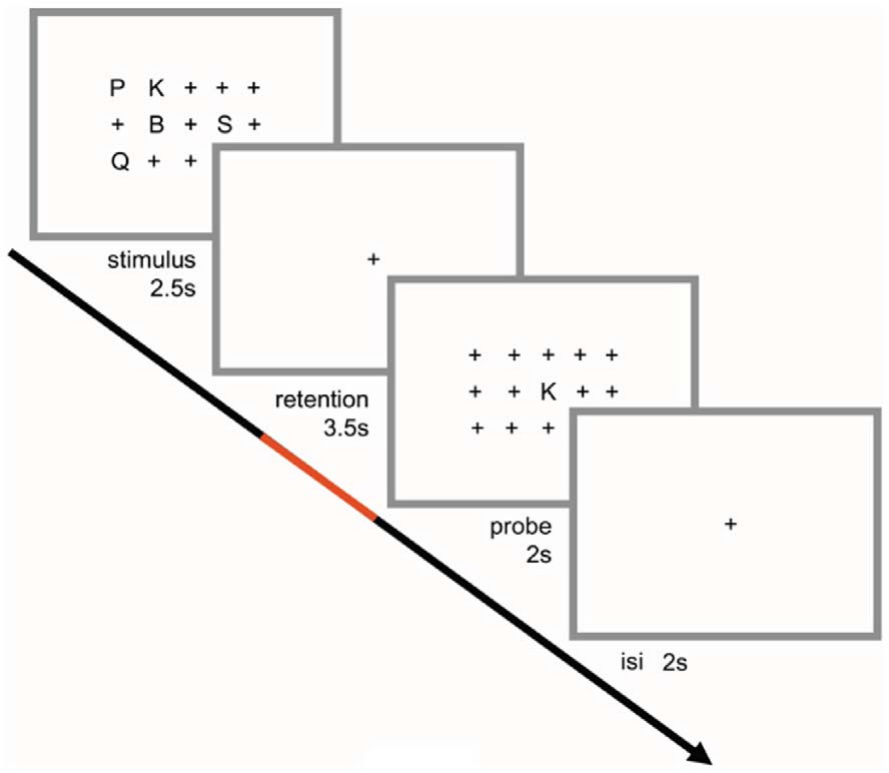
Illustration of experimental design. Sets of either 2 or 5 consonants were presented for 2.5 s (stimulus period) and had to be retained in memory for 3.5 s (retention period). After the retention period a probe letter was shown for 2 s (retrieval period). Subjects indicated by button press whether the probe was part of the stimulus. The red line indicates the last 2.5 s of the retention period that was used for EEG spectral analyses and for EEG-BOLD signal correlation analysis.

We used a Sternberg WM task in which the encoding of memory items, retention and retrieval period are temporally separated [5], [14], [36]. At the onset of each trial, an array of 15 items, arranged in three rows, appeared in black font at the centre of a white background (Fig. 1). The item array covered a visual angle of 6°×3°. The stimulus consisted of sets of either 2 or 5 consonants (from now on: ss2 and ss5). The remaining items were plus signs (+). The position of the consonants was counterbalanced across trials. The plus signs were used as fillers to ensure that the physical size and the visual content of the stimulus were the same, irrespective of the set size. The stimulus array remained on screen for 2.5 s, followed by a fixation cross presented for 3.5 s in the middle of the screen (retention period). After the retention period, a probe letter (also embedded in an item array of plus signs) was shown for 2 s (retrieval period). The inter-stimulus period consisted of a fixation cross and was randomly varied to minimize preparatory activity (range: 1800–2500 ms; mean: 2000 ms). Each load condition consisted of 40 trials. The *baseline condition* consisted of a fixation star (*) and was presented in six blocks, each with a duration of 24.5 s. Subjects were instructed to indicate by button press (‘yes/no’ forced choice decision task) whether the probe was part of the stimulus. Response button assignment (‘right/left’) was counterbalanced across subjects.

The stimuli were projected via a LCD projector onto a screen placed in front of the MR scanner. Subjects viewed the stimuli via a mirror attached to the MR-coil. Responses were made via a fibre-optics response button box (Lumina LP-400, Cedrus Corporation, San Pedro, USA). Stimulus delivery and response registration was controlled by Presentation (Neurobehavioral Systems Inc., Albany, CA, USA). To become familiar with the task, the subjects were given a short practice version (with different stimuli) of the task outside the scanner.

### Data acquisition

The simultaneous EEG was recorded using a 64-channel EEG cap (FMS, Munich, Germany). Electrode positions included all 10–20 system electrodes and the additional electrodes Afz, Fpz, FCz, CPz, POz, Oz, Iz, F5/6, FC1/2/3/4/5/6, FT7/8/9/10, C1/2/5/6, CP1/2/3/4/5/6,TP7/8/9/10, P5/6, PO1/2/9/10, OI1/2 plus two electrodes below the outer canthus of each eye and two electrocardiogram (ECG) electrodes. One ECG electrode was placed on the sternum and the other close to the heart. All channels were sampled at 5 kHz with 32 mV input range using a MR-conditioned amplifier (high pass filters: DC; low pass filters: 70 Hz) (Brainproducts, Munich, Germany). O1/2 and Fp1/2 were placed 5% more laterally for even coverage [37]. Impedances for over 98% of the electrodes were kept below 20 kOhm. The few electrodes with high impedances (between 20–50 kOhm) nonetheless delivered reliable EEG-signal [38]. Electrode F1 served as recording reference.

### EEG data and statistical analysis

EEG data were analyzed offline using the Brain Vision Analyzer Software (version 1.05, Brainproducts, Munich, Germany). The data were corrected for MR imaging gradient and ECG artefacts by template subtraction [39], [40]. After artefact correction, data were digitally bandpass filtered (0.5–50 Hz, slope: 24 dB/octave) and downsampled to 256 Hz. Artefacts related to eye movements, muscles, and (remaining) ECG were corrected using an ICA (provided by S. Enghoff, http://www.cnl.salk.edu). ECG related components were defined by time delay parameters as implemented in Brain Vision Analyzer [39], [40]. After artefact removal, data were transformed to the average reference [41]. The ECG channels were not included for further EEG analyses. Data with remaining artefacts exceeding ±150 µV in any channel were automatically rejected before averaging.

The spectral analysis focused on the last 2.5 s of the retention period (i.e., 2.5 s data segments), because it has been demonstrated previously that WM related alpha band power effects are suppressed in the first second of the retention period [4], [14]. For each of these segments a Fast Fourier Transformation (FFT) was computed (Hanning window: 10%), and the resulting power density values were averaged across segments, separately for the low and high workload conditions. The number of data points in these 2.5 s segments was automatically extended to the next higher power of 2 by zero padding, resulting in a frequency resolution of 0.25 Hz.

To compare the group results with previous studies using EEG spectral power, we calculated an index of global spectral power for our data [42]. For this purpose the root mean square (RMS, i.e. the square-weighted mean over the scalp) of spectral band power was calculated across channels and then plotted on topographical maps. Spectral analysis was performed for the following six frequency bands: theta (5–7 Hz), alpha1 (8–10 Hz), alpha2 (10–13 Hz), beta1 (13–20 Hz), beta2 (20–30 Hz), and gamma (30–40 Hz). In addition, EEG results were shown for single electrodes, which are known to be modulated by WM. For theta, we selected electrode Afz [5] and for alpha electrode O2 [23]. The load independent- and load dependent EEG analysis was performed as follows:

1. *Load independent analysis*: EEG power during the retention period:

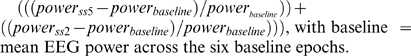

2. *Load dependent analysis*: The power in trials with ss5 was compared to trials with ss2 (‘ss5–ss2’) for each frequency point. Load effects were calculated as follows:

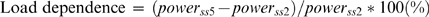

The more exploratory load independent- and dependent EEG effects were assessed by one sample t-tests (one-tailed, p<0.05 and p<0.01, respectively, uncorrected for multiple tests). However, load dependent EEG effects were also computed with the false discovery rate (FDR, p<0.05) correction approach [43], [44].

We used standardized low resolution brain electromagnetic tomography (sLORETA) to localize the generators of the scalp EEG power spectra for the contrast ‘ss5–ss2’. The sLORETA solution space is restricted to the cortical grey matter in the digitized MNI atlas with a total of 6239 voxels at 5 mm spatial resolution [45]. A spatial over-smoothing of 10^−4^ (variance smoothing  = 1, i.e. a single common variance for all variables) was chosen for the LORETA transformation matrix. Since sLORETA explicitly takes into account that scalp electric potentials are determined up to an arbitrary additive constant, the final sLORETA solution is independent of the electrical reference used. We calculated tomographic sLORETA images corresponding to the estimated neuronal generators of brain activity [46]. The EEG signal was first localized by sLORETA and then bandpassed for the same frequency bands as those for the spectral analysis. Statistical analysis was performed through multiple voxel-by-voxel comparisons using common non-parametric permutation tests, i.e. a non-parametric randomization statistic (SnPM) for functional brain imaging based on a bootstrapping approach to correct for multiple testing [47]. The core idea upon which the SnPM methodology is based can be attributed to Fisher's permutation test [48]. The significance threshold was based on a permutation test with 5000 randomizations. Unless stated otherwise, the t-values displayed correspond to p<0.05 (corrected for multiple comparisons using SnPM). Results were plotted onto a standard MRI template as described in detail elsewhere [45].

### FMRI data analysis

MRI data was acquired on a 3.0 T (GE Healthcare, Milwaukee, WI, USA) whole-body scanner. For fMRI, 558 scans sensitive to BOLD contrast with 33 axial slices covering the whole brain were acquired with a T2*-sensitive multi-slice echo planar imaging (EPI) sequence (repetition time  = 1.815 s; echo time = 32 ms; field of view  = 22 cm; image matrix  = 64×64; voxel size  = 3.44×3.44×3.8 mm^3^; flip angle  = 75°). The first 4 scans were discarded to allow for equilibration effects. Participants were fitted with earplugs. Particular care was taken to stabilize with vacuum cushions and custom made padding. Functional MRI data pre-processing and statistical analysis was done using SPM5 (Wellcome Department of Imaging Neuroscience, London, http://www.fil.ion.ucl.ac.uk/spm). The data were first motion corrected and the images were then normalized using a 4^th^ degree B-Spline interpolation method to match the Montreal Neurological Institute (MNI) EPI template. Two of eighteen subjects were excluded from the analysis due to major head movements (i.e., translation >1.5 mm) during scanning. Finally, functional volumes were resampled to isotropic 3 mm^3^ voxels and spatially smoothed with a 9 mm full width at half maximum isotropic Gaussian kernel. Functional images were temporally high-pass filtered with a cut-off period of 128 s, and serial correlations were accounted for by using an autoregressive model of the first order. No slice time correction was applied, because it has been demonstrated for event-related designs that slice time correction results in poor fMRI model fits and thus in type 2 errors [49]. Stimulus presentation was triggered automatically by the first slice acquisition of functional images.

### Statistical fMRI data analysis

Statistical fMRI analysis at the individual subject level was performed using the general linear model (GLM), as implemented in SPM5. Separate regressors were constructed for each phase of the task (encoding, retention, retrieval) and convolved with the hemodynamic response function for each of the two load conditions (ss2 and ss5). In addition, the frequency EEG regressors for the retention interval (see section *Statistical EEG-fMRI data analysis*) were included in the design matrix. A random effect second-level analyses was calculated for each task phase. Results were shown with both directions for each contrast (i.e., ‘retention (ss5 + ss2) - baseline’ and ‘baseline - retention’) at a corrected voxel threshold of p<0.05 using family-wise error correction [50], [51] and a cluster threshold of p<0.01 (k = 27 resampled voxels, corrected for multiple comparisons using Monte Carlo simulations with 1000 iterations, http://www2.bc.edu/~slotnics/scripts.htm [52]).The cluster threshold method was applied to control for the overall type I error.

For the load dependent fMRI analysis we calculated the contrasts ‘ss5–ss2’ and ‘ss2–ss5’. Results were shown at p<0.005 (corrected for multiple comparisons using the FDR). Both correct and incorrect trials were merged for the statistical analysis, because the overall accuracy was high (mean over all set sizes: 90.1%). Results are reported in the MNI coordinate system (Table 1).

**Table 1:** Summary of significant (p<0.005, FDR corrected) BOLD activations for the contrast ‘ss5–ss2’.

anatomical area	hemisphere	Brodmann	MNI coordinates			voxel
		area	x,y,z {mm}			(t-value)
cerebellum	left		−30	−63	−33	5.5
middle frontal g.	left	9	−48	21	33	5.46
thalamus	left		−12	−6	12	5.4
superior temporal g.	left	22	−54	15	−3	5.31
cerebellum	left		−39	−72	−33	4.9
inferior parietal lobe	left	40	−42	−51	48	4.85
nucleus caudatus	left		−18	−15	21	4.82
pons	left		−6	−42	−30	4.67
middle frontal g.	left	10	−33	48	12	4.67
lentiform nucleus	left		−9	6	−6	4.65
cerebellum	left		−9	−69	−12	4.65
parahippocampal g.	left		−30	−18	−15	4.65
precuneus	left	7	−21	−63	45	4.59
superior parietal lobe	left	7	−30	−54	48	4.58
inferior frontal g.	left	9, 44	−54	6	21	4.43
precentral g.	left	6	−39	−6	63	4.26
superior temporal g.	left	13	−54	−42	18	4.16
cerebellum	right		36	−66	−30	5.81
inferior frontal g.	right	47	33	21	−6	5.48
middle frontal g.	right	9, 46	48	36	33	5.17
inferior parietal lobe	right	39	33	−57	39	5.05
parietal lobe	right		27	−48	33	4.96
(para-)hippocampus	right	27	30	−30	0	4.88
frontal lobe	right		27	−16	27	4.86
middle frontal g.	right	9	42	42	36	4.84
cerebellum	right		12	−45	−27	4.77
inferior frontal g.	right		21	33	0	4.58
fusiform gyrus	right		42	−39	−6	4.55
inferior frontal g.	right	45, 47	57	18	0	4.14
cerebellum	right		9	−63	−39	4.13
middle frontal g.	right	10	36	48	9	4.1

Activations are reported by anatomical area, hemisphere, Brodmann area, MNI coordinates, and t-value. Only clusters at a corrected cluster threshold of p<0.01 are reported. G.: gyrus.

### Statistical EEG-fMRI data analysis

To study load independent trial-by-trial EEG-BOLD signal correlations related to the retention period, first separate EEG regressors for each of the 40 trials from each load condition and frequency band were constructed from the 2.5 s retention interval. The EEG regressor was calculated by averaging frequency points in the band of interest (i.e., from 5–7 Hz for theta etc.) for the RMS of all channels. Next, the EEG regressors for the six frequency bands were introduced as *parametric modulators of the retention period* in the GLM design matrix, resulting in nine regressors per load condition (i.e., the three fMRI task phases and six EEG frequency bands regressors for the retention period). To test for the influence of shared variance, we also performed the same type of analysis for the three fMRI task phases and a single frequency (i.e., a total of 4 regressors per load condition). Because we found a strong overlap between the EEG-BOLD signal correlations patterns for the two analyses we will report only results from the analysis with 9 regressors.

For all frequency bands, EEG-BOLD signal correlations were considered significant at an uncorrected voxel threshold of p<0.001 (t = 3.1) and at a corrected cluster threshold of p<0.01 (k = 27 resampled voxels using Monte Carlo simulations). The same voxel threshold has been used in a recent EEG-fMRI WM study [23].

For the load specific analysis first load sensitive regions from the BOLD contrast ‘ss5> ss2’ were identified (FDR with p<0.005). Activated regions at this threshold served as a mask for further processing. Therefore, this analysis was *not* a correlation analysis and was also not performed in regions that showed EEG-BOLD signal correlations. For each activated region, contrast estimates for each load condition and each frequency band were extracted for all participants using MarsBar [[53]; version 37]. Paired two-tailed t-tests were performed between contrast estimates of the two load conditions in each frequency band. An illustration of the procedure is shown in supplementary Figure S1.

## Results

### Behavioural

Overall the response accuracy was high (ss2: 91.3±24.6%, ss5: 88.8±24.1%) and tended to decrease with memory set size (t = 1.7; *p* = 0.08). Longer reaction times were found for ss5 in comparison to ss2 (t = −5.15, *p*<0.001; ss2 = 970.9±356.6 ms, ss5 = 1136.3±385.4 ms), consistent with results from a recent EEG-fMRI WM study [23].

### EEG

**Figure 2:**
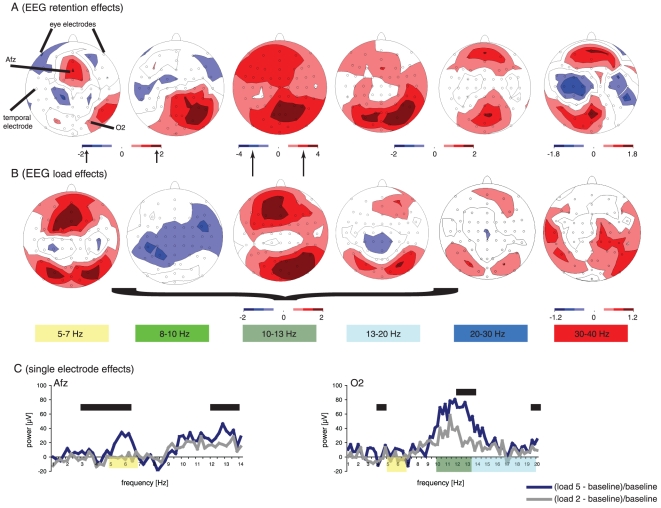
Illustration of EEG results during the retention interval. (**A**) Load independent topographical EEG effects. Results are reported for the following frequency bands: theta (5–7 Hz), alpha1 (8–10 Hz), alpha2 (10–13 Hz), beta1 (13–20 Hz), beta2 (20–30 Hz) and gamma (30–40 Hz). (**B**) Load dependent topographical EEG effects (‘(ss5–ss2)/ss2’). Significance values (t-tests, short arrow: p<0.05 and long arrow: p<0.01, uncorrected) for both analysis are indicated by a white-to-red (positive effects) and white-to-dark blue (negative effects) colour scale. (**C**) Results for single electrodes Afz and O2. For Afz, load 5 (dark-blue curve) exhibits stronger theta (yellow rectangle) power than load 2 (grey curve). For O2, alpha2 (light-green rectangle) and beta1 (light-blue rectangle) power is more enhanced for load 5 than for load 2. The black bars indicate significant effects at p<0.05 (corrected for multiple comparisons using the FDR). No significant load independent- or load dependent EEG effects occur at eye electrodes or at temporal (muscle) electrodes.

The topographical maps for the load independent analysis showed that theta power was significantly enhanced at frontal electrodes. By contrast, alpha1/2, beta1, and to weaker extent also beta2 and gamma, showed increased spectral power in parieto-occipital electrodes (Fig. 2A). Beta2 and gamma also exhibited significant (p<0.05) effects at frontal electrodes. No significant EEG power changes were observed at eye or typical muscle (i.e., temporal) electrodes for any of the frequency bands.

The load dependent analysis revealed predominately positive increases of power with set size in all frequency bands during the retention period, except in alpha1, as illustrated in Fig. 2B. The strongest load effects were observed in theta and alpha2. In addition, this analysis demonstrated a similar topographical power distribution as compared to the load independent analysis, especially in theta, alpha2, and beta1. Again, no significant effects occurred at eye- or muscle electrodes.

The single electrode analysis (Fig. 2C) indicated stronger load effects with higher workload in theta (electrode Afz), alpha2 and beta1 (electrode O2).

**Figure 3:**
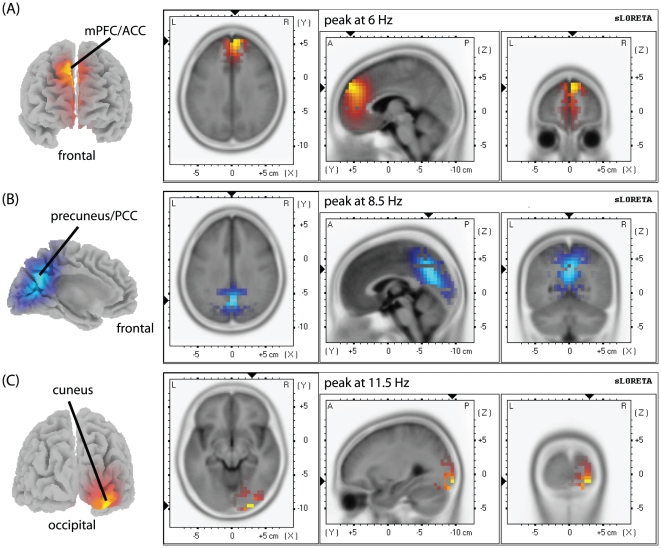
EEG source localization (sLORETA) activation maps for the contrast ‘ss5–ss2’. (**A**) Theta (5–7 Hz) band power increased (p<0.1) with load at the border of the MPFC and the ACC. (**B**) Alpha1 (8–10 Hz) showed decreased (blue) activity (p<0.05, corrected for multiple comparisons) at the border of the precuneus and the PCC with an increase of load. (**C**) Alpha2 (10–13 Hz) showed positive load modulations (p<0.05, corrected for multiple comparisons) in the right middle occipital gyrus (cuneus, BA 18). The t-values are plotted onto a MRI template.

Source localization for the contrast ‘ss5–ss2’ revealed a non-significant (p<0.1) increase of theta activity with load at the border of the MPFC and the ACC as shown in Fig. 3A. For alpha1, we observed significantly (p<0.05) decreased activity for the higher set size in the precuneus (Fig. 3B). For alpha2, we observed increased right lateralized activity in occipital regions (Fig. 3C).

### FMRI

**Figure 4:**
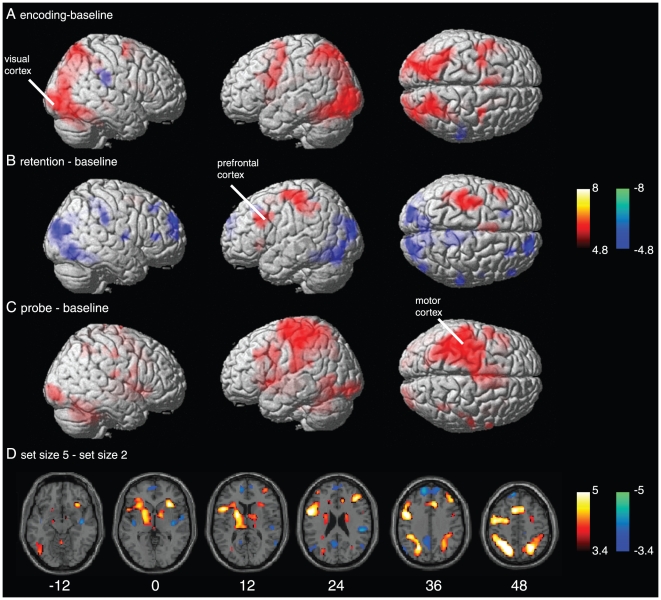
BOLD activation evoked by each task phase (encoding (A), retention (B), and retrieval (C)) contrasted against baseline (family-wise error corrected, p<0.05, red-yellow colour code). Activity for each phase is averaged across set size 2 and 5. Negative BOLD activations (i.e., ‘baseline - encoding’) are shown in a blue-green colour code. (**D**) BOLD activations for the contrast ‘ss5–ss2’ (red-yellow colour code) and ‘ss2–ss5’ (blue-green colour code). Activations are presented at p<0.005 (corrected for multiple comparisons using the FDR). Numbers indicate z-coordinates in MNI space.

Results for the fMRI analysis are displayed in Fig. 4. Encoding-related activity across both set sizes was found bilateral in occipital cortex (BA 18/19) as well as in parietal cortex (BA 7/40). Left dominant activations were found in the inferior frontal gyrus (IFG, BA 10/47), MPFC (BA 8/9) and the precentral gyrus (BA 6). Only weak negative activations (‘baseline > encoding’) were observed. The retention period activated the left MPFC and additionally the border of the postcentral gyrus (BA 3) and the parietal lobule (IPL, BA 40). The strongest negative activations (‘baseline > retention’) were predominantly found in the PCC, MPFC, and visual cortex. The BOLD response to the retrieval period was strongest in the left (contralateral to the response finger) primary motor cortex (BA 4) and bilaterally in occipital regions including the lingual gyrus (BA 17/18).

The load dependent analysis (i.e., contrast ‘ss5–ss2’) revealed bilateral activations in the PPC (BA 7), superior temporal gyrus (BA 22/42), IFG, superior frontal gyrus (SFG, BA 8), MPFC, thalamus, right insula, left lingual gyrus, DLPFC (BA 9/46), precentral gyrus, middle temporal gyrus (BA 21/37), putamen, and nucleus caudatus as shown in Fig. 4D. Furthermore, we found bilateral activations in the cerebellum. All significant activations for this contrast are listed in Table 1. The contrast ‘ss2–ss5’ activated predominantly the ACC (BA 32), PCC (BA 23/31), MPFC, and PPC.

### EEG-BOLD signal correlation analysis

**Figure 5:**
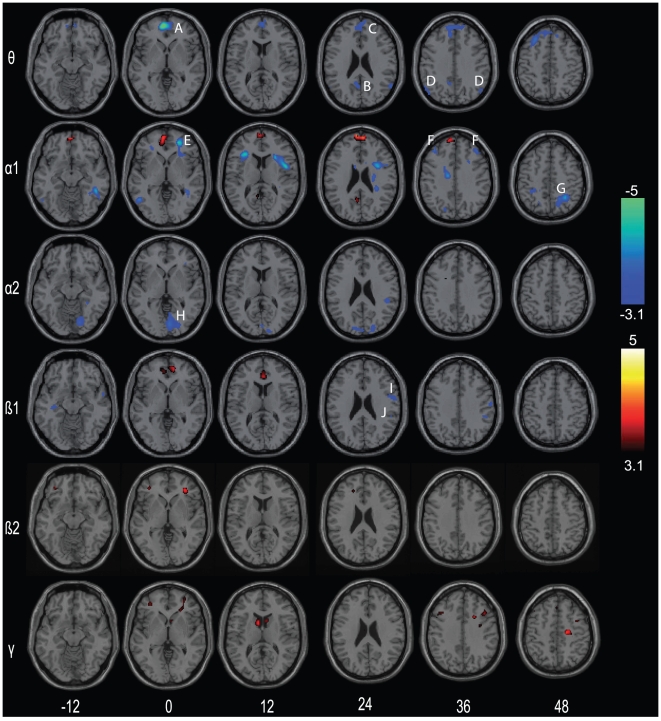
Illustrations of EEG-BOLD signal correlations during the retention interval. Positive (red) and negative (blue) correlations are shown for the following frequency bands: theta/θ: 5–7 Hz; alpha1/α1: 8–10 Hz; alpha2/α2: 10–13 Hz; beta1/β1: 13–20 Hz; beta2/β2: 20–30 Hz and gamma/γ: 30–40 Hz. All EEG-BOLD signal correlations are shown at an uncorrected voxel threshold of p<0.001 (t = 3.1) and a corrected cluster threshold of p<0.01. Numbers on the bottom indicate z-coordinates in MNI space. Capital letters in the different panels indicate the following brain regions: anterior cingulate cortex/ACC (A), posterior cingulate cortex/PCC (B), medial frontal gyrus/MPFC (C), posterior parietal cortex/PPC (D), inferior frontal gyrus (E), dorsolateral prefrontal cortex/DLPFC (F), precuneus (G), lingual gyrus (H), precentral gyrus (I), and border superior temporal gyrus and inferior parietal lobule (J). Correlations are shown for grey and white matter regions.

Figure 5 shows BOLD signal responses associated with EEG power fluctuation in different frequency bands during the retention period. Theta, alpha2 and -with one exception- also alpha1 demonstrated exclusively negative BOLD signal correlations, whereas beta2 and gamma showed only positive BOLD signal correlations. Specifically, negative theta-BOLD signal correlations occurred in ACC, MPFC, PCC and PPC. Alpha1 showed prominent negative BOLD signal correlations bilaterally in the PPC (including the precuneus), IFG (BA 47), DLPFC (BA 9), anterior insula, and middle temporo-occipital gyrus (BA 37). Positive correlations were found at the border of the ACC and MPFC, and in the PCC. For alpha2, negative correlations were observed in the lingual gyrus.

The EEG-BOLD correlations in higher frequency bands were overall spatially smaller and less extended than in lower frequency bands. Nevertheless significant correlations occurred in several grey matter regions as shown in Table 2 (for illustration purpose we additionally show positive beta2/gamma-BOLD signal correlations at a voxel threshold of p<0.005 (uncorrected) in supplementary Figure S2). Beta1 showed positive correlations only in ACC, whereas negative correlations were observed in the right precentral and postcentral gyrus, IFG (BA 44), and bilateral in the STG (BA 21/22). In beta2, positive correlations were visible in the insula, IFG and MPFC. Gamma correlations were positive in the nucleus caudatus (z = 12), DLPFC, SFG, precentral gyrus (BA 9), and MPFC.

**Table 2:** Illustration of grey matter high frequency BOLD signal correlations.

anatomical area	hemisphere	Brodmann	MNI coordinates	voxel
		area	x,y,z {mm}	(t-value)
**(A)**				
**positive**				
*cortical*				
anterior cingulate g.	right	24	6 36 6	3.12
**negative**				
*cortical*				
postcentral g.	right	2	66 −6 21	4.05
inferior frontal g.	right	44	54 3 21	3.98
precentral g.	right	6	63 −12 39	3.69
superior temporal g.	right	22	60 −15 0	3.92
superior temporal g.	left	21	−60 −12 −3	3.92
**(B)**				
**positive**				
*cortical*				
insula	right	13	36 18 11	4.28
inferior frontal g.	right	47	33 32 −8	3.93
middle frontal g.	left	11	−33 38 −12	3.76
**(C)**				
**positive**				
*cortical*				
precentral g.	left	9	−42 21 39	3.5
middle frontal g.	right	10	36 54 −6	3.43
precuneus	left	19	−39 −75 42	3.41
middle frontal g.	right	9	42 21 33	3.37
superior frontal g.	left	8	−3 27 54	3.14
middle frontal g.	right	6	42 1 54	3.11
*sub-cortical*				
nucleus caudatus	right		6 12 9	3.92
nucleus caudatus	left		−6 9 9	3.85

EEG-BOLD signal correlations for beta1 (**A**), beta2 (**B**) and gamma (**C**) are reported by anatomical name, hemisphere, Brodmann area, MNI coordinates, and t-value. Only clusters at a voxel threshold of p<0.001 (uncorrected) and at cluster threshold of p<0.01 (corrected) are reported. G.: gyrus.

### Load dependent effects

The load dependent analysis revealed that some brain regions demonstrated not only significant load BOLD effects (BOLD contrast: ‘ss5> ss2’) but also frequency bands related load effects (‘contrast estimate ss5> contrast estimate ss2’; Table 3). Specifically this analysis showed that theta load effects were exclusively positive, whereas alpha1 effects were exclusively negative (e.g. left DLPFC). Positive load effects in theta were visible in both cortical and sub-cortical brain regions such as the thalamus or putamen (see Discussion). In upper frequencies, load dependent effects were predominately positive and were most pronounced in the gamma band, i.e. in the MPFC, right SPL, and left DLPFC. In addition, sub-cortical structures, such as the thalamus, nucleus caudatus, and left putamen also exhibited positive load effects in gamma.

**Table 3:** Summary of load specific EEG frequency band effects in regions activated by the BOLD contrast ‘ss5–ss2’ (p<0.005, FDR corrected).

			*statistical comparisons for contrast estimates for individual frequency bands*					
region of interest	hemisphere	Brodmann	theta	alpha1	alpha2	beta1	beta2	gamma
		area						
cerebellum	left		+					
	right		+			+	+	
thalamus	left		+	-				
	right		+	-				
nucleus caudatus	right		+					+
putamen	left		+	-				
lingual g	left	17, 18	+	-				
superior frontal g	left	6, 8	+	-				
superior frontal g/medial frontal g	right	6	+	-				
superior frontal g./middle frontal g	right	6, 8, 46						+
inferior frontal g./superior temp g	left	47, 22				+		
	right		+			+	+	
inferior frontal g	right	47					+	
inferior frontal g/insula	right	47, 13						
middle frontal g	left	10	+				-	
	right			-				+
middle frontal g (DLPFC)	left	9, 46		--				+
	right							
middle frontal g/precentral g	left	6		-		+		
middle temp g	left	21						
middle temp g/hippocampal g	left	37						
superior parietal lobule	left	7	+	-				
	right		+					+

Positive EEG load effects (contrast estimate ss5> contrast ss2, t-test) are indicated by + (p<0.05); negative EEG load effects are indicated by -- (p<0.01) or - (p<0.05). For example, the left DLPFC shows positive load effects in gamma and negative load effects in alpha1. G.: gyrus, temp: temporal.

## Discussion

The main finding of our study is that both lower and higher frequencies correlated with the BOLD signal during the retention period of a WM task. In line with our hypothesis lower frequencies show primarily negative correlations, yet higher frequencies primarily positive correlations. We could also demonstrate that several brain regions display not only BOLD signal related load effects, but also frequency band related load effects.

### EEG effects

As a starting point for the load independent EEG-BOLD signal correlation analysis, first robust load independent EEG effects had to be identified. Our results revealed significant load independent EEG effects in all frequency bands as shown in Fig. 2. In addition, the load independent analysis revealed similar topographical maps to those derived for the load dependent analysis, especially in theta and alpha2, i.e. where the strongest load effects occur. For beta and gamma, we found weaker load independent EEG effects than those observed in lower frequency bands. Recently, it has been demonstrated that gamma EEG activity is susceptible to ocular muscular activity [54]. Therefore, one might ask whether gamma EEG effects are likely to reflect a global effect present in all channels and conditions, i.e. muscular artefacts, rather than neuronal effects. In order to reduce the likelihood of contamination of results by muscular artefacts, we used visual inspection and ICA to minimize artefact related to muscular activity, and transformed our data to the average reference and not to a single reference electrode (as performed by [54]), which reduces the chance of activation mislocalizations. Since the load independent (as well as load independent) analysis revealed that significant EEG effects in all frequency bands were neither present at any eye electrode nor at electrodes known to be affected by muscle activity, such as temporal electrodes (Fig. 2A and B), contamination of our results by muscular artefacts seems unlikely.

The finding of positive EEG load effects replicates earlier EEG/MEG findings for WM [4], [5], [8], [10], [14], [17], [23], [55]. For example, we found strong load effects (p<0.05, FDR corrected) at Afz (theta) and O2 (alpha2), i.e. at similar locations that have been reported in a recent study [23]. In addition, the sLORETA analysis reveals positive load effects in areas that have been previously described by other EEG/MEG studies of WM, e.g. in MPFC, ACC and parieto-occipital regions. There might be two reasons why we observed only partially (i.e., no significant load effects in gamma) significant EEG load effects. First, any potential effect could be partially confounded by scanning-related artefacts, namely by the MRI gradient and ECG artefact. However, the quality of our EEG data was good, and undistorted spectral map topographies were obtained after correction using a conventional analysis [39], [40] followed by an ICA approach [42], [56]. A recent EEG-fMRI to WM study used a similar experimental paradigm (Sternberg task with 0, 3, 5 and 7 letters as load conditions) and demonstrated similar load related EEG effects for measurements inside and outside the MR scanner [23]. We found that the proportion of artefacts related to muscles, eye movements, ECG, and MR gradient and signal components was around 1/5, i.e. 10–13 of the 62 independent components were classified as artefact components on the basis of the topographical distribution and IC signal, similar with the results reported by [57]. A more likely reason for weak load EEG effects could be the moderate load modulation of only five letters as used in our study. Several EEG/MEG and combined EEG-fMRI studies used six to eight items to be retained in memory [4], [14]. For this study, we opted for five letters as the high load condition in order to facilitate comparison with a large follow-up study, which will include children and adolescents. For the follow-up study we will use five letters as the high load condition, to ensure a high performance level across age span, which is one essential perquisite for comparing children and adolescent data to adult data.

Although we found EEG load effects in the majority of the investigated frequency bands in this study, we want to emphasize that these effects were not the main focus of the study, partly because we examined EEG-BOLD signal correlations during the retention period, and partly because the load dependent EEG-BOLD signal analysis was initiated from the BOLD- but not from the EEG contrast ‘ss5–ss2’.

### FMRI load effects

Our fMRI data confirm earlier fMRI findings of dominant WM activations in a fronto-parietal network [58]. It is important to note that our fMRI activations for the contrast ‘ss5–ss2’ are comparable to those reported for studies using longer retention periods, i.e. a 7 s retention period [23]. The event-related analyses demonstrate that the different short experimental phases elicit distinct activation patterns, e.g. the strongest activation occurred in the primary visual cortex during the stimulus period. During the retention period, activations were visible in typical areas of WM, i.e. in the DLPFC and PPC. The contrasts ‘baseline - retention’ and ‘ss2–ss5’ revealed similar deactivations in a second neuronal network. This network, where neural activity is greater during rest and suspended or deactivated during goal-directed behaviour, is called the *default mode network (DMN)*. It has been demonstrated by fMRI and EEG-fMRI studies that the DMN consists of the MPFC, insula, thalamus, ACC, PCC, PPC, occipito-temporal regions and the pre- and postcentral gyri [59], [60], [61], [62], [63]. Simultaneous EEG-fMRI studies have also demonstrated that predominantly lower EEG frequency bands show significant correlations with the BOLD signal during a mental arithmetic task [64], [65] or during rest [61], [66], [67], [68], [69], [70], [71], [72], [73]. Our findings are discussed in context of these EEG-BOLD signal interactions below.

### Are global EEG band measures valid tools for the investigation of EEG-BOLD signal correlations?

Global measures summarizing EEG activity across channels like RMS and global synchronization have the advantage of representing the full frequency band of interest without preselected electrodes, topographic components, or sources. Spectral RMS has been successfully used to investigate alpha-BOLD signal correlations during rest in healthy subjects [42]. However, averaging power over all channels might dilute task-related specific effects with a known topography. Thus, we also calculated theta and alpha2 EEG-BOLD signal correlations for electrodes that showed strong load effects in this and in other EEG/MEG studies, namely Afz (maximum load effect in theta) and O2 (maximum load effect in alpha2). In addition we selected electrode C3 to test whether alpha-BOLD signal correlations are likely to reflect the mu rhythm -which shows maximal amplitude over somatosensory cortices- rather than retention period related effects. First, supplementary Figure S3 demonstrates that the RMS represents local activities well, because the RMS approach yielded similar effects to the single-electrode approach in theta and alpha2. Therefore, our results are in line with other EEG-fMRI findings [23]. Second, alpha2-BOLD signal correlations do not reflect the mu rhythm, because C3-BOLD signal correlations were not manifested in somatosensory areas.

Of course, the RMS approach might be a more conservative way to isolate WM related effects with a known, constant topography than an ICA or single-electrode approach. However, the two latter approaches will work only for effects with a consistent (ICA) topography across subjects, such as in the theta band [17]. For example, in the alpha band it is not always possible to attribute a single ICA component to a load effect in all subjects (see [14]), and this problem is accentuated for higher frequency bands, because load effects are generally weaker at higher frequencies.

### EEG-BOLD signal correlation analysis: theta and alpha

The first main finding of the EEG-BOLD signal correlation analysis is that theta and alpha show predominantly negative correlations with the BOLD signal. Specifically, we demonstrate that theta showed exclusively negative correlations with BOLD signal in the DMN. These theta frequency-BOLD signal correlations are in line with a recent EEG-fMRI study that reported predominantly negative correlations in the DMN [23]. We thus conclude that BOLD effects during the contrast ‘ss2–ss5’ and ‘baseline - retention’ mainly reflect theta oscillations, because the BOLD activation pattern and the EEG-BOLD signal correlations showed a strong anatomical overlap (i.e., in the DMN) and were not present in other frequency domains (Fig. 5).

In contrast, alpha revealed dominant negative BOLD signal correlations in parieto-occipital regions including the precuneus (alpha1) or in the early visual cortex including the cuneus (alpha2) in line with other brain imaging studies [23]. During rest, many EEG-fMRI studies reported negative alpha-BOLD signal correlations [66], [67], [68], [71], [72]. Interestingly, the EEG-BOLD signal correlations comprises areas that are known to be involved in WM processing such as the MPFC, DLPFC and the PPC but also task irrelevant areas, i.e. the early visual cortex. The EEG-BOLD signal correlations in task irrelevant areas could be explained by the so-called inhibition hypothesis, which proposes that increased alpha power is related to top-down functional inhibition that may disrupt WM maintenance [9], [74].

The DLPFC and other frontal regions showed significant negative EEG-BOLD signal correlations in alpha1. The DLPFC is a core area of the WM network, because it is known that this region is activated as soon as manipulation of information is required, such as different load conditions [75]. It has been suggested [23] that the DLPFC might only synchronize at gamma, or alternatively shows little synchronization during the retention period, because the authors found no EEG-BOLD signal correlations in this region. Our results partly agree with this observation, as we found low frequency range correlations in the DLPFC in alpha1, i.e. close to the theta frequency band. This could indicate that the neuronal firing pattern might be desynchronized in alpha1, potentially as a result of reduced input from specific neurotransmitters. However, future studies are necessary to establish a link between EEG frequency power and the level of neurotransmitter concentration. In addition, our data lend support for the hypothesis proposed by [23], namely that the DLPFC synchronizes at gamma (i.e., positive gamma frequency-BOLD signal correlations).

In summary, our results demonstrate that (negative) low frequency-BOLD signal correlations are not only a marker for DMN [61], [66], [67], [68], [69], [70], [71], [72] activity but also for cognitive processing.

### EEG-BOLD signal correlation analysis: Beta and gamma

The second main finding of the EEG-BOLD signal correlation analysis showed exclusively significant positive (grey and white matter) correlations between beta2/gamma and the BOLD signal. Interestingly, these correlations were manifested focally than the correlations in lower frequency bands. For example, EEG-BOLD signal correlations for theta and alpha were localized in a large cortical network, e.g. in the DMN, whereas beta/gamma-BOLD signal correlations were restricted to only few cortical (and sub-cortical) regions. This finding is in line with earlier EEG findings that reported long-range (fronto-parietal) connections in lower frequency bands and short-range connections in the gamma band during a WM task [76].

It has been demonstrated that the BOLD signal mainly measures the hemodynamic correlate of neuronal LFP activity [77]. Our pattern of positive high frequency BOLD correlations is in accordance with studies using LFP [25], [26], [78] and a simple heuristic model [79]. For example, it has been found that in the visual cortex of macaque monkeys intracranial recordings of LFP correlated positively with the BOLD in the gamma range [77]. Similar, it has been reported that LFP gamma band activity decreased during visual stimulation when the BOLD signal was decreased [78]. Nonetheless, only few EEG-fMRI studies have reported positive correlations in higher frequencies, which are typically sparse and non-systematic [72] or subject-specific [70], [80].

We found that the DLPFC, MPFC, SFG, and IFG showed exclusively positive beta2 or gamma-BOLD signal correlations. This first indicates that the regions of the PFC must contain neurons that are modulated not only by slow but also by fast oscillatory activity during cognitive processing. During the retention period, spatially tuned elevated power in the beta and gamma band in LFP have been found in electrophysiological recordings from monkeys in parietal [81], extrastriate temporal cortex [82], and recently in the PFC [83]. Therefore, our results extend the latter finding that retention period related increased high frequency power at prefrontal electrodes is linked to the BOLD signal in human frontal regions such as MPFC (in addition in the IFG and SFG). The finding of positive beta/gamma-BOLD signal correlations in frontal regions is consistent with results from a EEG-BOLD signal study that reported positive correlations in their beta band (17–23 Hz) in the dorsal MPFC [72], and other DMN regions during rest. As shown in fMRI studies, cognitive control processes are supported by the DLPFC [84]. In addition, it has been reported that EEG gamma band activity increased with the increased cognitive control in the frontal regions in healthy subjects but not in schizophrenia subjects [85]. The authors suggested that the latter findings indicate a deficit in frontal cortical gamma band synchrony in patients with schizophrenia, which lead to the impairment in cognitive control. In humans, synchronization of cortical neurons in the gamma band seems to be important for a wide range of perceptual and cognitive processes [86], [87], [88], [89]. We suggest that EEG gamma band power in the DLPFC reflects cognitive control by neuronal synchrony, which is paralleled by higher oxygen demand. In addition, our load-specific analysis in higher frequency bands (and in theta) could indicate that neuronal synchrony is further elevated under higher cognitive demand (Table 3).

However, one might ask why we observed retention period related EEG-BOLD effects in low as well as in higher frequency bands in frontal regions. During long-term memory, there is evidence that the hippocampus and other regions of the cortex have dual oscillations, in which a theta oscillation is subdivided into subcycles by a high-frequency oscillation in the gamma range [90], [91]. It has been also demonstrated in model simulations that dual oscillations (i.e. subset of cells fire synchronously during a given gamma cycle) mediate the active maintenance of multiple short-term memories. Interestingly, there are about seven gamma cycles in a theta cycle, which could also explain why short-term WM is restricted to seven items [36]. Our results might indicate that the DLPFC contains neurons which allow the maintenance of short-term WM memories by dual oscillations.

In addition to frontal areas, the nucleus caudatus demonstrated positive gamma BOLD signal correlations. This region is part of a functional network involving the DLPFC and is activated during WM tasks [92], [93]. A destruction of the nucleus caudatus leads to the development of hyperactivity and attention disorders and to a deteriorated short-term memory [94], [95]. As for frontal regions, our data indicate that gamma oscillations in the nucleus caudatus are modulated during the retention interval and by higher cognitive load. This might suggest that the nucleus caudatus and frontal regions are coupled by neuronal synchrony of higher frequency bands.

The majority of studies relating electrophysiological brain signals to hemodynamics support the notion of a systematic relation between frequency and direction of the BOLD signal, in the sense that lower frequencies are coupled to BOLD signal decreases, whereas higher frequencies are linked to BOLD signal increases. Although our study lends support for this finding (i.e., negative theta/alpha2-BOLD signal correlations and positive beta2/gamma-BOLD signal correlations), we found both positive and negative effects in beta1, the latter of which were manifested in the precentral gyrus and at the border of the STG and IPL. These areas are also active during WM tasks [22], [29], [96]. Therefore, we suggest that within intermediate frequency bands -in our study alpha1 and beta1- the direction of EEG-BOLD signal correlations is not necessarily fixed but may vary across different brain regions.

### Intermediate conclusion

The investigation of the coupling between the BOLD signal and different EEG frequency bands enables the differentiation of neuronal activity based on their specific oscillatory activity. The load independent EEG-BOLD signal correlation analysis demonstrates that the interpretation of task-related neurovascular changes (i.e., the BOLD signal) is incomplete without incorporating the role of oscillatory activity. Our results indicate that lower and higher frequency bands are coupled to BOLD signal changes, extending findings from fMRI and EEG/MEG studies.

The next question is then whether EEG-BOLD interactions are further modulated under varying demanding conditions (contrast ‘ss5–ss2’). This will be discussed in the following section.

### Load dependent effects

The load specific analysis additionally revealed that some load sensitive areas (identified by the BOLD contrast: ‘ss5–ss2’) also exhibit frequency related load effects (Table 3).

Interestingly, theta, gamma and (mostly) beta demonstrated only positive load modulations, whereas alpha showed either no (alpha2) or negative (alpha1) load-effects. This is in line with human intracranial EEG findings of positive gamma load effects in the occipital lobe but also in other regions including the MPFC [22], and with several EEG/MEG studies, which found increased beta and gamma activity with WM load throughout the brain [9], [27], [28], [30], [31], [97], [98].

Our study adds significantly to these findings by showing that load dependence in some regions of the WM network is not only reflected by BOLD signal- or EEG/MEG changes alone, but by closely coupled EEG-BOLD signal changes. Our data also indicates that this coupling generally has opposite directions for low and higher frequencies, but that the coupling may also vary for a given frequency across different brain regions. For example, the left DLPC showed negative and positive load effects in different frequency bands. In contrast, the SPL showed exclusively positive load modulations in lower and higher frequency bands. These two general findings might indicate firstly that different neuronal populations must be engaged within the same cortical region operating at different frequencies. Second, it may suggest that negative EEG-BOLD signal correlations in lower frequencies do not automatically reflect processing of less cognitively demanding conditions, because we found negative theta-BOLD signal correlations during the retention period but positive load effects in (similar) frontal regions.

We also found load effects in sub-cortical regions such as the thalamus. Several studies demonstrated that in the thalamus alpha band power is positively linked to the BOLD signal during rest in healthy subject but also in patients with epilepsy [68], [99]. This indicates that simultaneous EEG-fMRI recordings can be used to examine cortical and sub-cortical regions, which can not easily be achieved by source localization, as source localization estimates sometimes have a spatial spread that is much larger than the actual underlying small, deep sources (i.e., there is ‘leaking’ of source power into regions surrounding the actual source). However, we want to emphasize that we do not claim that load effects in sub-cortical regions imply that these regions reflect the source of the signal but only that these regions show both load dependent BOLD and frequency bands effects.

One could argue that a load specific EEG-BOLD analysis should be either performed in regions that show retention period related BOLD effects or significant EEG-BOLD signal correlations. However, BOLD effects during the retention period differ substantially from the observed load specific BOLD effects (Fig. 4) and are limited to a small brain network. In addition, most of the EEG-BOLD signal correlations were found outside of the retention BOLD network. Other studies have also shown that EEG-BOLD signal correlations differ for individual frequency bands and spread to regions which are not observed with the EEG or the BOLD signal alone [23], [24]. For example, load effects in theta are strongest at frontal electrodes; whereas EEG-BOLD signal correlations in this frequency band involve not only the PFC but involve also parietal and cingulate regions. Therefore, we suggest that restricting an analysis to only those regions activated by the retention period might limit the scope of a load specific analysis.

### Conclusion

Our data demonstrate that simultaneous EEG-fMRI recordings are a useful tool for investigating state-dependent brain activity, e.g. during the retention period of a WM task. We suggest that the observed state-dependent dynamics of positive and negative activity fits with the view of the brain being driven by momentary environmental demands. From the load independent analysis we conclude that the directions of EEG-BOLD signal correlations vary for EEG frequency bands. In addition we suggest that both negative and positive EEG-BOLD signal correlations can characterize the same area.

From the load dependent analysis we further conclude that the coupling of frequency band oscillations and the BOLD signal in task-related areas changes with cognitive load, i.e., predominantly positive load effects occur across frequency bands (theta, beta, and gamma). In summary, our results indicate that low as well as high oscillatory activity is linked to neuronal activity during cognitive demanding processes.

## Supporting Information

Figure S1 Illustration of the steps performed for the load dependent EEG-BOLD signal analysis. The first step (panels on the top) is the calculation of the BOLD signal contrast ss5-ss2 (p<0.005, FDR corrected). The second step is the extraction of the contrast estimates for the different frequency bands and load conditions in the activated region (some areas are highlighted by capital letters A-F in the upper panel). For each region statistical comparisons (paired t-tests) are calculated (step 3, panel in the middle) which results in a colour-coded significance map (step 4, panel on the bottom). For example the left DLPFC (red arrow) shows a negative load effects (contrast estimates set size 5>contrast estimate set size 2) in alpha1 as well as a positive load effect in the gamma band (i.e., blue bar in the panel on the bottom).
(1.94 MB EPS)

Figure S2 Group averaged grey- and white matter beta2-BOLD (A) and gamma-BOLD (B) signal correlations (p<0.005, uncorrected). Red areas indicating positive EEG-BOLD signal correlations.
(1.92 MB EPS)

Figure S3 (A) EEG-BOLD signal correlations (group results, p<0.001, uncorrected) for the RMS and electrodes Afz, C3 and O2. Blue areas indicating negative EEG-BOLD signal correlations, red areas indicating positive EEG-BOLD signal correlations. (B) Statistical differences between the RMS and the single electrode approach (paired t-tests, k>15 voxel). For example, the two bottom panels on the left indicate regions in which Afz theta-BOLD signal correlations are stronger than RMS-BOLD signal correlations (green colour code).
(18.19 MB EPS)
